# A Case Report of a Thyrotropin-Secreting Pituitary Macroadenoma

**DOI:** 10.7759/cureus.27216

**Published:** 2022-07-25

**Authors:** Batoul Atwi, Zeinab Melhem, Boshra Yaacoub, Mariam Awada, Zeinab Issa

**Affiliations:** 1 Endocrinology and Diabetes, Lebanese University, Faculty of Medical Sciences, Beirut, LBN; 2 Internal Medicine, Rafik Hariri University Hospital, Beirut, LBN; 3 Endocrinology and Diabetes, Al-Zahraa University Hospital, Beirut, LBN

**Keywords:** thyroid adenoma, pituitary adenoma. brain tumor, tsh-secreting adenoma, tshoma, hyperthyroidism

## Abstract

Thyroid-stimulating hormone (TSH)-secreting pituitary adenoma is a rare case that is characterized by high or inappropriately normal thyrotropin levels along with an increase in thyroid hormones that lead, in most of the patients, to signs and symptoms similar to those of hyperthyroidism problems. Its diagnosis and management are still challenging.

A 65-year-old male patient presented to the emergency department for palpitations. He was firstly misdiagnosed due to incomplete lab tests. After a full workup, he was found to have TSH-secreting pituitary adenoma and referred to trans-sphenoidal surgery for macroadenoma excision. Currently, he is maintained on somatostatin analogue and methimazole.

This is the second case report of TSHoma in Lebanon with signs and symptoms of thyrotoxicosis. Usually, the clinical features of TSHomas vary between patients which makes the confirmation of diagnosis more difficult. Surgery is still the first line of treatment with the addition of encouraging effects of medical therapy consisting of somatostatin analogues.

## Introduction

Thyroid-stimulating hormone-secreting pituitary adenomas (TSHomas) are uncommon, accounting for less than 1% of all pituitary tumors [1]. Recently, its prevalence has increased to about 1-3% of all pituitary adenomas [1,2]. It is characterized by the autonomous secretion of TSH regardless of the negative feedback effect of thyroid hormones [3]. Majority of these patients present with symptoms of hyperthyroidism due to the overstimulation of TSH leading to high levels of T3 and T4 hormones [3]. Their clinical management has changed significantly owing to earlier diagnosis and initiation of somatostatin analogue therapy [3]. However, surgical excision is still considered to be the first-line treatment of TSHomas [4]. In this report, we introduce a case of a 65-year-old male patient diagnosed as having TSHoma as the second case reported in Lebanon.

## Case presentation

A 65-year-old male patient presented to the emergency department complaining of palpitations. On physical examination, diffuse and symmetrically enlarged thyroid was noted. Eye examination was normal, but there were no records of visual field examination, as well as no noticed skin abnormalities except for warm moist skin. Besides, he was found to have sinus tachycardia on ECG with a pulse rate of 146 bpm. He had no past medical history and wasn’t taking any home medication as well.

The patient was referred to a cardiology outpatient clinic for the evaluation of an underlying cardiac disease. He underwent echocardiography ultrasound imaging and treadmill stress test which both showed normal results, and he was prescribed bisoprolol 5mg once daily. The patient continued to experience episodes of palpitations, so he was advised to visit an endocrinologist to evaluate for endocrine dysfunction that might explain his tachycardia.

The endocrinologist he visited first asked for a TSH level blood test which turned out high: 9 mU/l (RR 0.27 - 4.2 mU/l). He was considered in a hypothyroid state based on the high TSH level only and prescribed levothyroxine treatment. However, the patient continued to have frequent prolonged palpitations, and subsequently, he sought another opinion. This time, he was asked for complete thyroid function tests which revealed elevated TSH as well as high FT3 and FT4 levels. Thus, the patient’s diagnosis was completely changed to secondary hyperthyroidism.

Workup

Laboratory tests that were done upon diagnosis and their results are listed in Table 1.

**Table 1:** Results of laboratory tests done upon diagnosis of patient’s secondary hyperthyroidism TSH: Thyroid-stimulating hormone; FSH: Follicle-stimulating hormone; LH: Luteinizing hormone; ACTH: Adrenocorticotropic hormone; FT3: Free triiodothyronine; FT4: Free thyroxine.

Lab test	Result	Normal range
TSH	10.15 mU/l	0.27 - 4.2 mU/l
FT3	7.86 pmol/l	2.58 - 5.44 pmol/l
FT4	28.63 pmol/l	12 - 22 pmol/l
Prolactin	15 ng/ml	1.61 - 18.77 ng/ml
Growth hormone	0.50 ng/ml	<7.00 ng/ml
FSH	5.94 mIU/ml	1.40 - 18.10 mIU/ml
LH	6.29 mIU/ml	1.50 - 9.30 mIU/ml
Antiperodixase antibodies	4.0 IU/ml	<9.0 IU/ml
ACTH	26 pg/ml	10 - 48 pg/ml
Cortisol	117 ng/ml	50 - 250 ng/ml
Testosterone	1049 ng/dl	225 - 972 ng/dl

**Figure 1:**
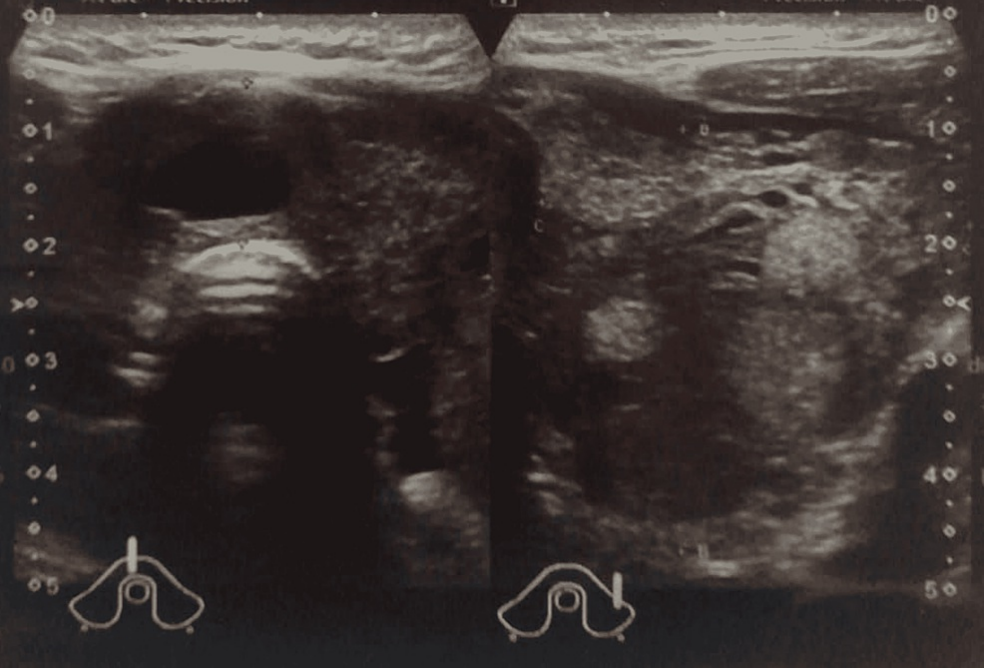
Patient’s thyroid ultrasound upon diagnosis showing multi-nodular goiter with the largest nodule measuring 24x22x21 mm in the lower pole of the left thyroid lobe.

Ultrasound of the thyroid was done, as shown in Figure 1, revealing an enlarged thyroid gland with features of multinodular goiter with the largest nodule located in the lower pole of the left thyroid lobe measuring 24 x 22 x 21 mm. As concerning his nodules, fine needle aspiration to the latter largest nodule was found to return with benign cytology.

The patient also underwent an MRI of the brain which showed a pituitary macroadenoma tumor. This latter MRI was done abroad thus we couldn’t reach images except for the radiologist’s written report only that showed an enlarged sella turcica with associated pituitary enhancing mass, with no measurements written, occupying the left side of the pituitary gland and causing upward convexity with subsequent displacement of the pituitary stalk to the contralateral right side. Associated invasion of the ipsilateral left cavernous sinus is noted with encasement of the intracavernous portion of the internal carotid artery. The subsequent invasion of the root of the sphenoid sinus is noted. There is an upward extension of the tumor to the suprasellar cistern in close proximity to the left side of the optic chiasm. However, there is no significant optic chiasmal compression.

Management

The initial treatment given to the patient consisted of somatostatin analogue (20 mg of Sandostatin LAR injection followed by a second injection dose after one month), along with methimazole (Tapazole 5mg; three tablets/day). TSH was repeated after two months showing 13.6 mIU/l (normal: 0.3-4.0 mIU/l) with normal FT4 level of 1.1 ng/dl (normal: 0.78-1.9 ng/dl). Two weeks later, surgical intervention was conducted via a trans-sphenoidal approach to extract the macro-adenoma.

Post-operatively, our patient was placed again on a combination of medical therapy consisting of anti-thyroid medication, oral corticosteroid, beta-blocker and somatostatin analogue as shown in Table 2. MRI was repeated after four months of surgery that revealed interval regression of size of macroadenoma with residual adenoma still noted measuring 1 x 1.4 cm.

**Table 2:** Medical therapy taken by the patient currently

Drug	Description
Methimazole (tapazole)	5 mg: Three tablets/day reduced to two tablets a day based on the improvement of FT4 result (16.37 pmol/L)
Prednisone (Cortancyl)	5 mg: One tablet/day, was stopped based on Cortisol Synacthen test (8:30 am: 167 nmol/L, 9:00 am: 397 nmol/L, 9:30 am: 455 nmol/L)
Bisoprolol (Concor)	5 mg: One tablet/day
Somatostatin analogue (Sandostatin LAR)	20 mg: One injection/month for 10 months, shifted to 30 mg monthly

Two months later after surgery, TSH level becomes 8.56 mU/l (normal: 0.27-4.2 mU/l) with FT4 level of 16.37 pmol/L (normal: 12-22). The patient’s follow-up of lab tests showed stability of his thyroid hormones’ levels, pituitary hormones’ levels, along with the stability of his pituitary adenoma with no increase in size on serial MRI imaging.

**Figure 2:**
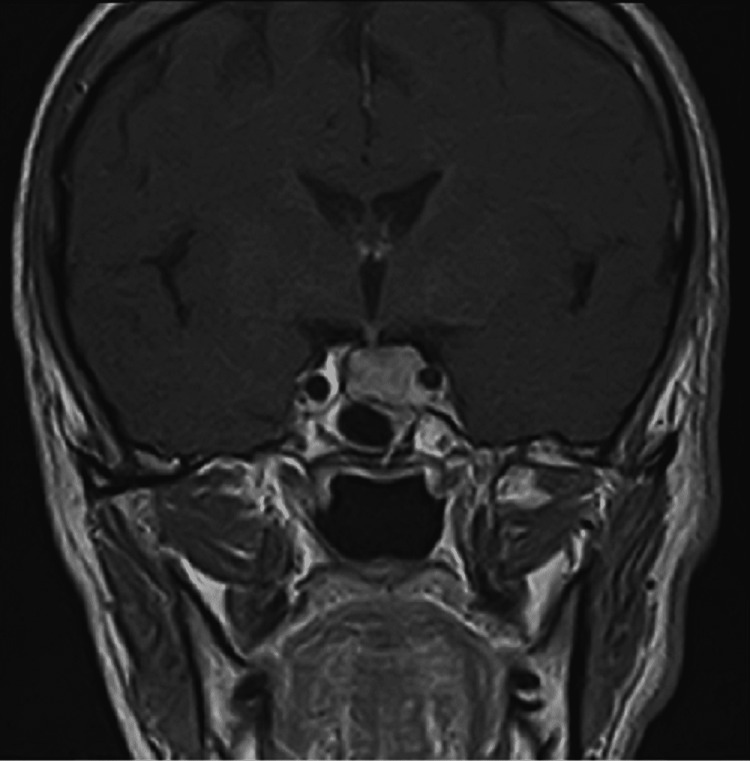
Latest MRI done of coronal cut image showing an isointense pituitary lesion to the grey matter of 1cm x 1.3cm invading the left cavernous sinus with 180 degrees encasement of the cavernous portion of the left carotid artery.

The latest MRI seen in Figure 2 showed an isointense pituitary lesion to the grey matter of 1cm x 1.3cm invading the left cavernous sinus with 180 degrees encasement of the cavernous portion of the left carotid artery. This result presented no change in size compared with previous images after the surgery. The neurosurgeon was reconsulted for re-doing the trans-sphenoidal surgery who thought the latter will not be a curative approach, and so the patient continued on his current medical regimen.

## Discussion

It is well known that in primary hyperthyroidism (e.g., Graves’ disease, toxic nodular goiter, or a toxic adenoma), serum TSH levels are usually suppressed, often to undetectable levels [5]. The presence of elevated serum TSH levels along with elevated thyroid hormones in the setting of hyperthyroidism suggests a secondary or central origin [6]. In such settings, the presence of a pituitary tumor on brain imaging, suggests the rare condition of pituitary thyrotropin hypersecretion as the cause of hyperthyroidism [3]. TSHomas are a rare type of pituitary tumors (accounting for 0.5-3% of all functional pituitary adenomas) and a rare cause of hyperthyroidism (<1%) [6]. Males and females are equally affected and the mean age of diagnosis is in the 40s [6]. TSHomas are usually benign adenomas arising from a monoclonal expansion of neoplastic thyrotropes [2]. They have been reported in the pituitary as well as in ectopic sites such as the pharynx [7]. TSHoma has many types based on the secretory profile (only TSH, TSH and growth hormone (GH), multiple pituitary hormones) [5].

Most of the time, TSH secreting adenomas present with signs and symptoms of hyperthyroidism [3]. Because of its rarity, data collected on this condition are relatively few, and because the diagnosis is usually delayed, these tumors are mostly diagnosed as macroadenomas, preventing an effective cure and leading to more local and systemic comorbidities [8]. So some new researches throw light on the etiopathogenesis, the diagnosis and the treatment of such an exceptional disease [3]. The diagnosis has been facilitated after the development of ultra-sensitive TSH immunometric assays and dynamic testing, such as T3 suppression tests and TRH, which are beneficial in differentiating TSHomas from syndromes of thyroid hormone resistance [9]. The first line treatment of TSHomas is pituitary neurosurgery [10]. The medical therapy of TSHomas depends mainly on somatostatin analogues, such as octreotide and lanreotide, which showed to decrease TSH levels in most of the patients effectively and thus rendering the patient in the euthyroid state by normalization of FT3 and FT4 [9].

Our case presented a 65-year-old man diagnosed with TSH secreting pituitary adenoma after he first complained of palpitations and thereafter found to have an enlarged thyroid on ultrasound. His workup then showed high TSH, FT3, and FT4 along with a pituitary tumor on MRI. Although it shares many aspects with similar cases published worldwide, our case has some different features. Despite being a macroadenoma, our patient didn’t experience any local mechanical compression effect on optic chiasm leading to visual field defects. He also didn’t develop any clinical complications such as vision loss or hormonal dysfunction and barely noticed his condition by the palpitations he experienced. Moreover, the fact that this is the second reported case of TSHoma in Lebanon makes our case a valuable one to be published [11].

A very important lesson to be learnt from this case report is to interpret thyroid function tests (TFTs) as a whole unit and not to treat a patient based on TSH results only. Keeping in mind that abnormal TSH can be a consequence of a central cause, although uncommon, is a must and ordering FT3 and FT4 in front of an abnormal TSH level is necessary to avoid misdiagnosis.

## Conclusions

In conclusion, this is the second case report in Lebanon of TSH-secreting pituitary adenoma. The mean age of diagnosis of TSHoma is around 41-45 years. Yet, it is worth mentioning that keeping this condition in our differential diagnosis as physicians facing other causes of hyperthyroidism is important regardless of the patient’s age. Failure to recognize the presence of a TSHoma may lead to negative consequences, such as an inappropriate thyroidectomy decision that may cause the pituitary tumor to expand more. Therefore, early diagnosis along with proper management prevents undesirable neurological and endocrinological complications, and so provides better consequences and a cure rate.
